# Discovery in space of ethanolamine, the simplest phospholipid head group

**DOI:** 10.1073/pnas.2101314118

**Published:** 2021-05-24

**Authors:** Víctor M. Rivilla, Izaskun Jiménez-Serra, Jesús Martín-Pintado, Carlos Briones, Lucas F. Rodríguez-Almeida, Fernando Rico-Villas, Belén Tercero, Shaoshan Zeng, Laura Colzi, Pablo de Vicente, Sergio Martín, Miguel A. Requena-Torres

**Affiliations:** ^a^Centro de Astrobiología, Consejo Superior de Investigaciones Científicas–Instituto Nacional de Técnica Aeroespacial “Esteban Terradas”, 28850 Madrid, Spain;; ^b^Osservatorio Astrofisico di Arcetri, Istituto Nazionale de Astrofisica, 50125 Florence, Italy;; ^c^Observatorio Astronómico Nacional, Instituto Geográfico Nacional, 28014 Madrid, Spain;; ^d^Star and Planet Formation Laboratory, Cluster for Pioneering Research, RIKEN, Wako 351-0198, Japan;; ^e^ALMA Department of Science, European Southern Observatory, Santiago 763-0355, Chile;; ^f^Department of Science Operations, Joint Atacama Large Millimeter/Submillimeter Array Observatory, Santiago 763-0355, Chile;; ^g^Department of Astronomy, University of Maryland, College Park, MD 20742;; ^h^Department of Physics, Astronomy and Geosciences, Towson University, Towson, MD 21252

**Keywords:** astrochemistry, ethanolamine, molecular clouds, prebiotic chemistry, cell membranes

## Abstract

Cell membranes are a key element of life because they keep the genetic material and metabolic machinery together. All present cell membranes are made of phospholipids, yet the nature of the first membranes and the origin of phospholipids are still under debate. We report here the presence of ethanolamine in space, ${NH}_{2}{CH}_{2}{CH}_{2}$OH, which forms the hydrophilic head of the simplest and second-most-abundant phospholipid in membranes. The molecular column density of ethanolamine in interstellar space is N = (1.51± 0.07)$\times \;10^{13}\;{cm}^{-2}$, implying a molecular abundance with respect to $H_{2}$ of $\left(0.9-1.4\right)\;\times \;10^{-10}$. Previous studies reported its presence in meteoritic material, but they suggested that it is synthesized in the meteorite itself by decomposition of amino acids. However, we find that the proportion of the molecule with respect to water in the interstellar medium is similar to the one found in the meteorite ($10^{-6}$). These results indicate that ethanolamine forms efficiently in space and, if delivered onto early Earth, could have contributed to the assembling and early evolution of primitive membranes.

## Significance

The detection of ethanolamine (${NH}_{2}{CH}_{2}{CH}_{2}$OH) in a molecular cloud in the interstellar medium confirms that a precursor of phospholipids is efficiently formed by interstellar chemistry. Hence, ethanolamine could have been transferred from the proto-Solar nebula to planetesimals and minor bodies of the Solar System and thereafter to our planet. The prebiotic availability of ethanolamine on early Earth could have triggered the formation of efficient and permeable amphiphilic molecules such as phospholipids, thus playing a relevant role in the evolution of the first cellular membranes needed for the emergence of life.

**Fig. 1.**
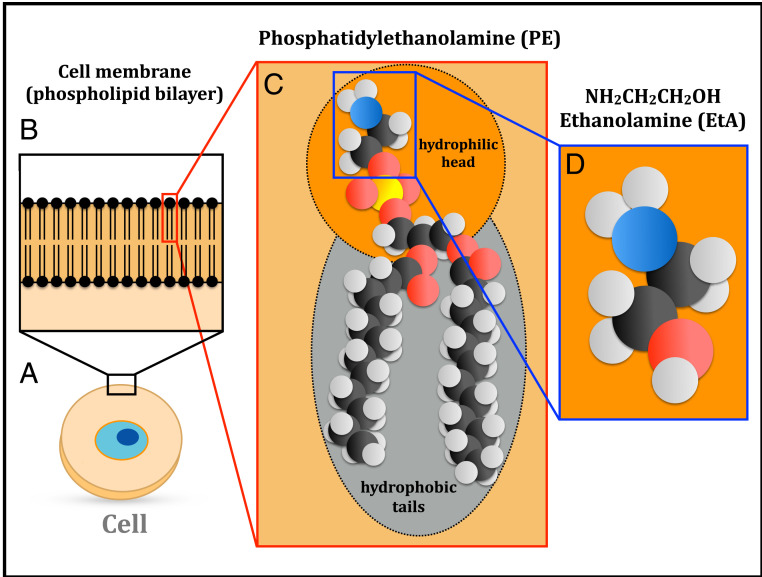
Structure of cellular membranes. (*A*) Schematic view of a cell. (*B*) Zoom-in view of the cell membrane, formed by a phospholipid bilayer. (*C*) Three-dimensional structure of the phospholipid PE, formed by a hydrophilic head composed of EtA, a phosphate group linked to glycerol, and two hydrophobic fatty-acid tails (black, red, blue, and white balls denote carbon, oxygen, nitrogen, and hydrogen atoms, respectively). (*D*) EtA, the molecular species detected in space and reported in this work.

Life is based on three basic subsystems: a compartment, a metabolic machinery, and information-bearing molecules together with replication mechanisms (1, 2). Among these key elements, compartmentalization is a fundamental prerequisite in the process of the emergence and early evolution of life (3, 4). Indeed, cellular membranes encapsulate and protect the genetic material, as well as enable the metabolic activities within the cell. The membranes of all current cells are made of a bilayer of phospholipids (Fig. 1 *A* and *B*), which are composed of a polar hydrophilic head (an alcohol phosphate group combined with a head group such as ethanolamine [EtA], choline, or serine) and two nonpolar hydrophobic tails (hydrocarbon chains derived from fatty acids), as depicted in Fig. 1*C*.

The process through which the first phospholipids were formed remains unknown. Initial work proposed that phospholipids could be synthesized under possible prebiotic conditions (5–7), but the availability of the precursor molecules on early Earth was questioned (3, 8). Alternatively, the building blocks of phospholipids could have been delivered from space. A broad repertoire of prebiotic molecules could have been provided to the early Earth through the bombardment of comets and meteorites (9, 10). Laboratory impact experiments (11, 12) have demonstrated that a significant fraction of the prebiotic molecules in comets and meteorites can survive both passage through the planetary atmosphere and the impact on the surface.

In particular, some structural parts of phospholipids are known to be present in meteorites, such as fatty acids, alcohols, and phosphonic acids (10, 13, 14). The glycerol phosphate group has been shown to be synthesized in irradiation experiments of interstellar ice analogs (15, 16), which supports the idea that they can form in space. Regarding the different head groups of phospholipids, EtA (also known as glycinol or 2-aminoethanol, ${NH}_{2}{CH}_{2}{CH}_{2}$OH; Fig. 1*D*) is the simplest one, and it forms the second-most-abundant phospholipid in biological membranes: phosphatidylethanolamine (PE) (see Fig. 1*C*). In addition, EtA has been proposed as a direct precursor of the simplest amino acid, glycine (${NH}_{2}{CH}_{2}$COOH), in simulated archean alkaline hydrothermal vents (17), considered as one of the likely environments for the origin of life (18).

EtA has been found in the Almahata Sitta meteorite (19), yet its origin is not known. A possible chemical formation route was proposed to be the thermal decomposition of amino acids under specific unusual conditions in the parent asteroid. This would limit the availability of EtA in the early Earth for the formation of phospholipids and thereafter of cell membranes. Another possibility is that EtA is formed from smaller interstellar precursors. However, the detection of EtA in the interstellar medium (ISM) has remained so far elusive (20).

## Results

**Fig. 2.**
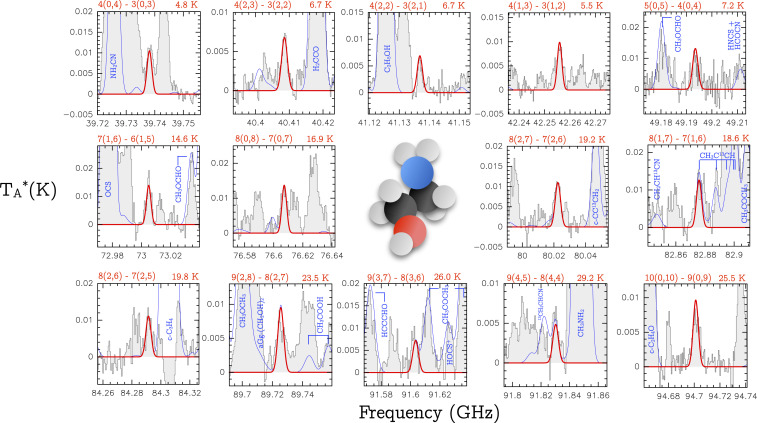
Unblended or slightly blended transitions of EtA toward the G+0.693–0.027 molecular cloud. The quantum numbers involved in the transition are indicated in the upper left of each panel, and the energies of the upper level are indicated in the upper right. The red thick line depicts the best LTE fit to the EtA rotational transitions. The thin blue line shows the expected molecular emission from all of the molecular species identified in our spectral survey, overplotted to the observed spectra (gray histograms). The three-dimensional structure of EtA is shown in the center of the figure; black, red, blue, and white balls denote carbon, oxygen, nitrogen, and hydrogen atoms, respectively.

We have detected EtA toward the molecular cloud G+0.693–0.027 (hereafter G+0.693), located in the SgrB2 complex in the Galactic Center, as shown in Fig. 2. This region is one of the most chemically rich reservoirs of molecules in the galaxy, with a plethora of organic species detected (21–25). The extremely rich gas-phase chemical composition of this region is due to erosion of the ice mantles of interstellar dust grains by large-scale low-velocity (<20 km$\cdot s^{-1}$) shocks (26) induced by a collision between massive molecular clouds (27). For the typical (intermediate) densities of G+0.693 of a few $10^{4}\;{cm}^{-3}$ (27), the emission is subthermally excited, yielding very low *T*_ex_ in the range of 5 to 15 K (21, 22). Since only low-energy molecular transitions are excited, the density of molecular lines is substantially lower than in hotter sources such as massive molecular hot cores or low-mass hot corinos, alleviating the problems of line blending and line confusion. This, along with the effect of shock-induced desorption of interstellar ices, makes G+0.693 an excellent target for the detection of new molecular species in the ISM.

We analyzed the molecular data of a high-sensitivity unbiased spectral survey carried out with the Institut de Radioastronomie Millimétrique (IRAM) 30-m and the Yebes 40-m radiotelescopes. Detailed information about the observations is presented in *Materials and Methods*. The identification of the rotational transitions of EtA was performed using the SLIM (Spectral Line Identification and Modeling) tool within the MADCUBA package (28). We predicted the synthetic spectrum of EtA under the assumption of local thermodynamic equilibrium (LTE) conditions. Among the numerous (23,655) transitions of EtA that fall in the spectral range covered by the survey, only tens of them are expected to be excited considering the low *T*_ex_ measured in G+0.693 (*T*_ex_ ∼5 to 15 K) (21, 22).

**Fig. 3.**
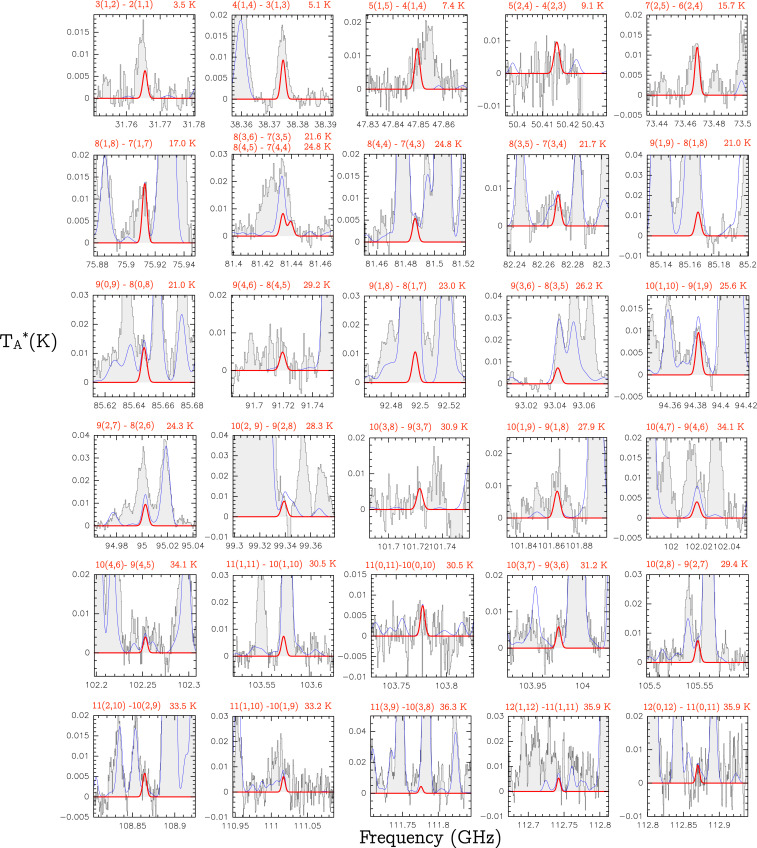
Transitions of EtA that appear blended in the observed spectra of G+0.693. All of them have line intensities >5 mK, according to the best LTE fit described in the text. The quantum numbers involved in the transition are indicated in the upper left of each panel, and the energies of the upper level are indicated in the upper right. The red thick line depicts the best LTE fit obtained fitting the EtA rotational transitions shown in Fig. 2. The thin blue line shows the predicted molecular emission from all of the molecular species identified in our spectral survey, overplotted to the observed spectra (gray histograms).

We have detected the 45 brightest transitions of EtA, as predicted by the LTE simulation (with line intensities $T_{A}^{*}\;>$ 5 mK), 14 of which appear either unblended or slightly blended with emission from other molecules. These transitions are shown in Fig. 2, and their spectroscopic information is provided in Table 1. The remaining 31 transitions are consistent with the observed spectra but appear blended with brighter emission lines from other molecular species already identified in this molecular cloud (see below). These transitions are shown in Fig. 3 and listed in Table 2.

**Table 1.** Spectroscopic information (rest frequency, Einstein coefficients [$A_{ul}$], and energy of the upper levels [$E_{up}$]) of the 14 unblended or slightly blended rotational transitions of EtA detected toward the G+0.693 molecular cloud (shown in Fig. 2)

Frequency, GHz	Transition	log$A_{ul}$, $s^{-1}$	$E_{up}$, K
39.7379429	4(0,4)–3(0,3)	−5.64603	4.8
40.4083769	4(2,3)–3(2,2)	−5.74549	6.7
41.1366268	4(2,2)–3(2,1)	−5.72208	6.7
42.2557255	4(1,3)–3(1,2)	−5.59088	5.5
49.1932727	5(0,5)–4(0,4)	−5.35979	7.2
73.0048603	7(1,6)–6(1,5)	−4.84093	14.6
76.6071016	8(0,8)–7(0,7)	−4.76916	16.9
80.0223886	8(2,7)–7(2,6)	−4.73500	19.2
82.8757878	8(1,7)–7(1,6)	−4.67140	18.6
84.2912932	8(2,6)–7(2,5)	−4.66443	19.8
89.7254251	9(2,8)–8(2,7)	−4.57750	23.5
91.6032870	9(3,7)–8(3,6)	−4.57780	26.0
91.8301065	9(4,5)–8(4,4)	−4.61854	29.2
94.7010488	10(0,10)–9(0,9)	−4.48695	25.5

**Table 2.** Spectroscopic information (rest frequency, Einstein coefficients [$A_{ul}$], and energy of the upper levels [$E_{up}$]) of the transitions of EtA that appear blended in the observed spectra of G+0.693 (see Fig. 3)

Frequency, GHz	Transition	log$A_{ul}$, $s^{-1}$	$E_{up}$, K
31.7653500	3(1,2)–2(1,1)	−6.00114	3.5
38.3746402	4(1,4)–3(1,3)	−5.71626	5.1
47.8493375	5(1,5)–4(1,4)	−5.40899	7.4
50.4153833	5(2,4)–4(2,3)	−5.39849	9.1
73.4680803	7(2,5)–6(2,4)	−4.85633	15.7
75.9126193	8(1,8)–7(1,7)	−4.78270	17.0
81.4338930	8(3,6)–7(3,5)	−4.74841	21.6
81.4392902	8(4,5)–7(4,4)	−4.80721	24.8
81.4863522	8(4,4)–7(4,3)	−4.80646	24.8
82.2698610	8(3,5)–7(3,4)	−4.73500	21.7
85.1653931	9(1,9)–8(1,8)	−4.62876	21.0
85.6467486	9(0,9)–8(0,8)	−4.62059	21.0
91.7192567	9(4,6)–8(4,5)	−4.62007	29.2
92.4964225	9(1,8)–8(1,7)	−4.52562	23.0
93.0416052	9(3,6)–8(3,5)	−4.55708	26.2
94.3821793	10(1,10)–9(1,9)	−4.49166	25.6
95.0023981	9(2,7)–8(2,6)	−4.49975	24.3
99.3388437	10(2,9)–9(2,8)	−4.43880	28.3
101.7229298	10(3,8)–9(3,7)	−4.42905	30.9
101.8639901	10(1,9)–9(1,8)	−4.39813	27.9
102.0183898	10(4,7)–9(4,6)	−4.45929	34.1
102.2528632	10(4,6)–9(4,5)	−4.45637	34.1
103.5718907	11(1,11)–10(1,10)	−4.36815	30.5
103.7762062	11(0,11)–10(0,10)	−4.36532	30.5
103.9759166	10(3,7)–9(3,6)	−4.39964	31.2
105.5481523	10(2,8)–9(2,7)	−4.35654	29.4
108.8642389	11(2,10)–10(2,9)	−4.31491	33.5
111.0166375	11(1,10)–10(1,9)	−4.28448	33.2
111.7757005	11(3,9)–10(3,8)	−4.29721	36.3
112.7422238	12(1,12)–11(1,11)	−4.25550	35.9
112.8698247	12(0,12)–11(0,11)	−4.25393	35.9

All transitions have line intensities >5 mK, according to the best LTE fit described in the text.

To confirm that the spectral lines detected at the frequencies of the transitions of EtA are not produced by any other molecule, we have performed an extensive search for molecular species in our spectral survey, which includes all of the species detected so far in the ISM (29), and all other species reported toward G+0.693 in previous works (21–25). The predicted contribution from all molecular species is shown with a blue solid line in Fig. 2, confirming that 14 transitions of EtA are either clean or not significantly contaminated by the emission from other molecules. We have used these 14 transitions to perform the LTE fit and to derive the physical parameters of the emission of EtA. We used the AUTOFIT tool of MADCUBA−SLIM, which finds the best agreement between the observed spectra and the predicted LTE model (see details in *Materials and Methods*). To perform the fit we have considered not only the emission of EtA but also the predicted emission from all of the species identified in the region (blue line in Fig. 2). The best-fitting LTE model for EtA gives a molecular column density of N = (1.51± 0.07)$\times \;10^{13}\;{cm}^{-2}$, an excitation temperature of $T_{ex}$ = 10.7 ± 0.7 K, and a velocity of $v_{LSR}$ = 68.3 ± 0.4 km$\cdot s^{-1}$ (the linewidth was fixed to 15 km$\cdot s^{-1}$; see details in *Materials and Methods*). The derived $T_{ex}$ and $v_{LSR}$ are very similar to those from other species previously analyzed in G+0.693 (21–25). To derive the abundance of EtA with respect to molecular hydrogen, we have used the $H_{2}$ column density inferred from observations of $C^{18}$O (26), obtaining a value in the range (0.9 to 1.4) × 10^−10^.

**Fig. 4.**
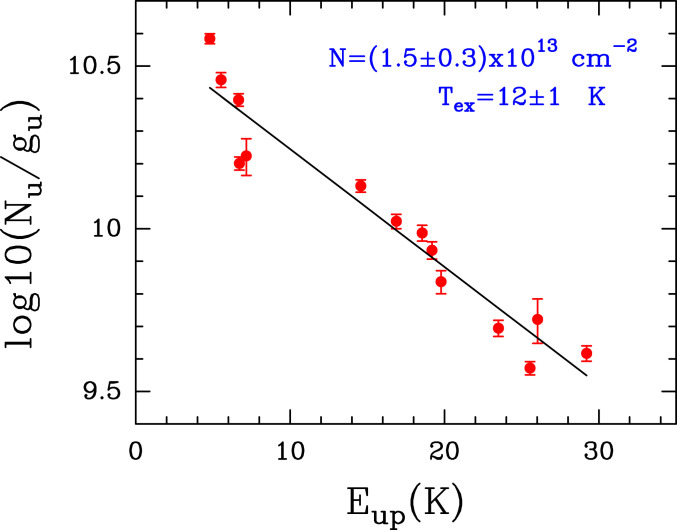
Rotational diagram of EtA. The analysis procedure is described in *Materials and Methods*. The red dots correspond to the 14 EtA transitions shown in Fig. 2 and Table 1. The black line is the best linear fit to the data points. The derived values for the molecular column density (N) and the $T_{ex}$, along with their uncertainties, are indicated in blue in the upper right corner.

We have also performed a complementary analysis using the rotational diagram method implemented in MADCUBA (see further description in *Materials and Methods*). Fig. 4 shows the rotational diagram obtained using the 14 EtA transitions from Fig. 2. We derived physical parameters fully consistent with the MADCUBA−AUTOFIT analysis: N = (1.5 ± 0.3)$\times 10^{13}\;{cm}^{-2}$, and $T_{ex}$ = 12 ± 1 K.

## Discussion

We report a clear detection in the ISM of EtA, a precursor of phospholipids, with a relatively high abundance ($10^{-10}$ with respect to molecular hydrogen). This detection adds to that of precursors of ribonucleotides (23–25) and amino acids (30, 31) in the ISM. The building blocks of the three subsystems of life could therefore have been synthesized by interstellar chemistry, being part of the natal material that formed the Solar System.

**Fig. 5.**
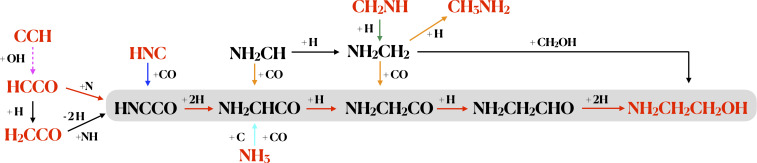
Summary of the chemical routes proposed for the formation of EtA in the ISM. The molecular species in red have been detected toward the G+0.693 molecular cloud. The gray shaded area corresponds to a hydrogenation chain. The chemical reactions indicated with colored arrows have been proposed in previous work: magenta (36), blue (38), orange (41), cyan (39), and green (41, 47). In black, we show the formation routes proposed in this work. The solid arrows indicate surface chemistry reactions, and dashed arrows denote gas-phase chemistry.

The formation routes of EtA in the ISM are, however, poorly known. Grain-surface formation of EtA has been demonstrated by laboratory experiments of ultraviolet irradiation of interstellar ice analogs (32). In these experiments, photolysis of $H_{2}$O:${CH}_{3}$OH:${NH}_{3}$:HCN ices with a 20:2:1:1 mixture yields EtA as well as other prebiotic species such as the amino acids glycine, alanine, and serine. However, the detailed routes that result into the formation of EtA are still not understood. We discuss here several possible chemical pathways for the formation of EtA in the ISM, which are summarized in Fig. 5.

To our knowledge, the only route proposed in the literature (33, 34) is the hydrogenation chain of HNCCO on dust-grain surfaces (see gray shaded area in Fig. 5). HNCCO could be formed on grains by N addition to ketenyl (HCCO) (34). HCCO is rarely found in the ISM, with only two detections reported toward the cold dark clouds Lupus-1A and L483 (35). We have also searched for HCCO in G+0.693, and tentatively detected it. The details about this detection are provided in *Materials and Methods*. We obtain a column density of $∼0.5\times 10^{13}\;{cm}^{-2}$, a factor ∼3 lower than that of EtA. HCCO is expected to be a highly reactive radical on dust grains. This would result in a low ice abundance of HCCO, and consequently also in the gas phase, due to shock-induced sputtering desorption. Alternatively, the amount of HCCO detected toward G+0.693 might have been produced directly in the gas phase through the reaction CCH + OH → HCCO + H proposed by ref. 36, since CCH is highly abundant in this cloud (37).

HNCCO could also be formed on dust grains from ketene ($H_{2}$CCO), after two hydrogen abstractions, and reaction with the imine radical NH (Fig. 5). G+0.693 presents a variety of imines with relatively high abundances (22, 23, 37), which confirms that imine radicals are available on grain surfaces. This route is plausible since ketene is abundant toward G+0.693 (21) with a column density of N = 2.9 $\times 10^{14}\;{cm}^{-2}$, a factor of ∼20 larger than that of EtA. Alternatively, the formation of HNCCO on grains could proceed as proposed by ref. 38 (Fig. 5) through the combination of HNC and CO, species expected to be abundant on grain surfaces.

The subsequent hydrogenation of HNCCO can form ${NH}_{2}$CHCO (Fig. 5). This species might also form through other surface-chemistry routes. Ref. 39 proposed a barrierless reaction between ${NH}_{3}$, CO, and atomic C (Fig. 5). Given that three-body reactions are less efficient that two-body reactions, this route could contribute to the formation of ${NH}_{2}$CHCO only if a relatively high abundance of atomic C is available. Since it has been observed that the abundance of C is indeed large in Galactic Center molecular clouds, around half of that of CO (40), and considering that C is expected to be highly reactive, this route might be indeed viable in G+0.693. We note that the barrierless ${NH}_{2}$CH + CO reaction proposed by ref. 41 might also contribute to the formation of ${NH}_{2}$CHCO (Fig. 5).

The hydrogenation of ${NH}_{2}$CH could yield the ${NH}_{2}{CH}_{2}$ radical, which might be a key precursor of EtA. In recent laboratory experiments (42, 43) of the nonenergetic formation of simple amino acids and sugars under prestellar conditions, intermediate radicals such as ${NH}_{2}{CH}_{2}$ and ${CH}_{2}$OH are efficiently formed in the hydrogenation reactions toward methylamine (${CH}_{3}{NH}_{2}$) and methanol (${CH}_{3}$OH). These radicals represent the structural units of EtA and, hence, this species could be produced by the nondiffusive reaction between ${NH}_{2}{CH}_{2}$ and ${CH}_{2}$OH on the surface of dust grains (Fig. 5). Similar radical–radical reactions have been proposed as viable routes to form other complex species in the ISM (44–46). ${NH}_{2}{CH}_{2}$ is expected to be present on the dust grains of G+0.693 since it is an intermediate product between methanimine (${CH}_{2}$NH) and methylamine (${CH}_{3}{NH}_{2}$) (41, 42, 47), two species that are abundant in G+0.693 (22).

Moreover, ${NH}_{2}{CH}_{2}$ could react with CO, as proposed by ref. 41, to form ${NH}_{2}{CH}_{2}$CO, which can be hydrogenated to form EtA (Fig. 5). Unfortunately, there is no rotational spectroscopy available for HNCCO, ${NH}_{2}$CHCO, or ${NH}_{2}{CH}_{2}$CO, so we cannot search for any of these possible precursors of EtA in the G+0.693 spectral survey.

The penultimate step of the hydrogenation chain that results into EtA is aminoacetaldehyde (${NH}_{2}{CH}_{2}$CHO). The rotational spectra of this species have been studied theoretically by ref. 48, although the accuracy of the predicted frequencies (∼0.2%) is still not high enough for any reliable identification in the ISM. Our detection of EtA toward G+0.693 makes ${NH}_{2}{CH}_{2}$CHO a promising species for future interstellar searches and should motivate new laboratory work to obtain its microwave rotational spectrum with higher accuracy.

The detection of EtA reported in this work with an abundance of $∼10^{-10}$ with respect to $H_{2}$ enables a rough comparison with the concentration of this species measured in meteoritic material (19). Considering that the abundance of water in the ISM is of the order of $10^{-4}$ (49), the EtA/$H_{2}$O abundance ratio measured in G+0.693 is of the order of $10^{-6}$. The Almahata Sitta meteorite, where EtA was detected (19), has been classified as a ureilite with an anomalously high fraction of other materials, the enstatite chondrites (EC) being the most abundant (50). Interestingly, EC meteorites have recently been proposed as the origin source of most of Earth’s water (51). Therefore, meteorites such as Almahata Sitta could have simultaneously delivered to Earth not only water but also prebiotic chemicals such as EtA. From the concentration of EtA measured in the Almahata Sitta meteorite of 20 ppb (19), and the average concentration of water in EC meteorites (∼7,500 ppm) (51), we derive a meteoritic EtA/$H_{2}$O abundance ratio of 3 $\times 10^{-6}$. This value is consistent with that derived in the ISM. Although isotopic analysis of EtA would be needed to confirm its interstellar origin in meteorites, our results suggest that phospholipid precursors such as EtA formed in the ISM could have been stored in planetesimals and minor bodies of the Solar System, to be subsequently transferred to early Earth.

Once EtA was available on Earth’s surface, it could form phospholipids (in particular PE; see Fig. 1*C*) under plausible early Earth conditions, as proposed by ref. 6 and confirmed by prebiotic experiments (7). It is commonly assumed that the first cell membranes could have been composed of amphiphilic molecules such as fatty acids/alcohols, which are chemically simpler than phospholipids (3, 8). However, the availability of EtA in an early Earth could have enabled the progressive replacement of fatty acids/alcohols by more efficient and permeable amphiphilic molecules such as phospholipids. In this scenario, the protocells could have been able to incorporate from the environment the precursor molecules required to start the synthesis of RNA and eventually other polymeric molecules (52, 53) needed for the first replicative and metabolic processes of life. This has important implications not only for theories of the origin of life on Earth but also on other habitable planets and satellites anywhere in the universe.

## Materials and Methods

### Astronomical Observations.

We have analyzed a high-sensitivity spectral survey of the molecular cloud G+0.693–0.027 conducted with the Yebes 40-m telescope (Guadalajara, Spain) and the IRAM 30-m telescope (Granada, Spain). The observations were centered at the equatorial coordinates of G+0.693: RA(J2000) = 17 h 47 m 22s, DEC(J2000) = −28° 21′ 27”.

#### Yebes 40-m telescope.

The observations were carried out with the Yebes 40-m telescope located in Yebes (Guadalajara, Spain), during six observing sessions in February 2020, as part of the project 20A008 (Principal Investigator I.J.-S.). We used the new Nanocosmos Q-band (7-mm) high-electron-mobility transistor receiver that enables ultrabroad-band observations in two linear polarizations (54). The receiver is connected to 16 fast Fourier transform spectrometers with a spectral coverage of 2.5 GHz and a spectral resolution of 38 kHz. The final spectra were smoothed to a resolution of 251 kHz, corresponding to a velocity resolution of 1.7 km$\cdot s^{-1}$ at 45 GHz. We covered a total spectral range from 31.075 GHz to 50.424 GHz. The position switching mode was used, with the reference position located at (−885″, +290″) with respect to G+0.693 (24, 27). The telescope pointing and focus were checked every 1 or 2 h through pseudo-continuum observations toward VX Sgr, a red hypergiant star near the target source. The spectra were measured in units of antenna temperature, $T_{A}^{*}$, since the molecular emission toward G+0.693 is extended over the beams (55). The noise of the spectra depends on the frequency range, reaching values as low as 1.0 mK, while in some intervals it increases up to 4.0 mK. The half-power beam width (HPBW) of the telescope is 48″ at 36 GHz.

#### IRAM 30-m telescope.

We have carried out a spectral survey at 3 mm using the IRAM 30-m telescope. The observations were performed in two observing runs during 2019: 10 to 16 April and 13 to 19 August, from project numbers 172-18 (Principal Investigator J.M.-P.) and 018-19 (Principal Investigator V.M.R.). We used the broad-band Eight MIxer Receiver (EMIR) and the fast Fourier transform spectrometers in FTS200 mode, which provided a channel width of ∼200 kHz. The final spectra were smoothed to a 609 KHz, i.e., a velocity resolution of 1.8 km$\cdot s^{-1}$ at 100 GHz. The full spectral coverage is 71.770 to 116.720 GHz. The telescope pointing and focus were checked every 1.5 h toward bright sources. The spectra were also measured in units of antenna temperature, $T_{A}^{*}$. The noise of the spectra (in $T_{A}^{*}$) is 1.3 to 2.8 mK in the range 71 to 90 GHz, 1.5 to 5.8 mK in the range 90 to 115 GHz, and ∼10 mK in the range 115 to 116 GHz. The HPBW of the observations vary between 21.1″ and 34.3”. The position switching mode was used in all observations with the off position located at (−885″, +290″) from the source position.

### SLIM Molecular Line Fitting.

The identification of the molecular lines was performed using the SLIM tool of the MADCUBA package.* SLIM solves the radiative transfer equation, as described in detail in ref. 28, and generates the expected synthetic spectra of the molecular species under the assumption of LTE conditions. SLIM implements a stand-alone HyperSQL database (http://hsqldb.org/) that contains the spectral line catalogs of the Jet Propulsion Laboratory (https://spec.jpl.nasa.gov/) (JPL) (56) and the Cologne Database for Molecular Spectroscopy (CDMS) (https://cdms.astro.uni-koeln.de/) (57, 58).

For the case of EtA, we have used the spectroscopic entry 61004 (version September 2003) of the JPL database, based on different laboratory works (59–61). The value of the partition function (Q) at the temperatures of the fit ($T_{ex}∼$ 11 K) has been interpolated from the values reported in the JPL catalog in the logQ–logT plane, using the two adjacent temperatures: Q (9.375 K) = 254.2935 and Q (18.75 K) = 716.8160.

To derive the physical parameters from the molecular emission, we have used the AUTOFIT tool of SLIM (28), which performs a nonlinear least-squares fitting of simulated LTE spectra to the observed data. It uses the Levenberg–Marquardt algorithm (62, 63), which combines the gradient descent method and the Gauss–Newton method to minimize the $\chi^{2}$ function.

For the analysis of EtA, we fixed the linewidth (full width at half maximum, FWHM) to 15 km$\cdot s^{-1}$, which reproduces well the observed spectral profiles of the EtA transitions and is consistent with those measured for other molecules in the region (22, 24, 25). We note that the upper energy levels ($E_{up}$) of the transitions used in the analysis span a range between 4.8 and 29.2 K, allowing us to determine the $T_{ex}$ of the emission. The molecular column density (N), $T_{ex}$, and the velocity ($v_{LSR}$) were left as free parameters. The best-fitting LTE model gives N = (1.51± 0.07)$\times 10^{13}\;{cm}^{-2}$, $T_{ex}$ = 10.7 ± 0.7 K, and $v_{LSR}$ = 68.3 ± 0.4 km$\cdot s^{-1}$.

To compute the relative molecular abundance with respect to molecular hydrogen we have used the value of the $H_{2}$ column density inferred from observations of $C^{18}$O, 1.35 $\times 10^{23}\;{cm}^{-2}$ (26). We have assumed a 20% error uncertainty in the determination of the $H_{2}$ column density and propagated the error accordingly. The EtA molecular abundance falls in the range (0.9 to 1.4) × 10^−10^.

### Rotational Diagram Method.

The rotational diagram is calculated following the standard procedure (64) implemented in MADCUBA (28). For the case of optically thin emission the velocity integrated intensity over the linewidth (FWHM = 15 km$\cdot s^{-1}$), W (in kelvin kilometers per second), is converted into the column density in the upper level of the transition $N_{up}$ (in centimeters^−2^) using the expression

$$N_{up}=8\pi k\nu^{2}W/\left(hc^{3}A_{ul}\right),$$ [1]

where k is the Boltzmann constant, the frequency of the transition, h is the Planck’s constant, c is the speed of light, and $A_{ul}$ is the Einstein coefficient of spontaneous emission from the upper level u to lower level l. Then, the level population derived for all observed transitions can be combined to determine the total molecular column density, N (in centimeters^−2^), and the $T_{ex}$ (in kelvin) through the equation

$$\text{log}\left(N_{up}/g_{up}\right)=\text{log}\left(N/Q\left(T_{ex}\right)\right)-\text{log}\left(e\right)\times E_{up}/\left(kT_{ex}\right),$$ [2]

where $g_{up}$ and $E_{up}$ are respectively the statistical weight and energy (in kelvin) of the upper levels of the transitions and Q is the partition function.

Fig. 4 shows the plot of log($N_{up}$/$g_{up}$) versus $E_{up}$ for all of the unblended or slightly blended transitions (see Fig. 2 and Table 1). The error bars indicate the uncertainty of the velocity integrated intensity (ΔW), which is derived using the expression

$$\Delta W=rms\times {\left(\Delta v/FWHM\right)}^{0.5}\times FWHM,$$ [3]

where rms is the noise of the spectra and Δv is the spectral resolution of the data in velocity units. The coefficients of the straight line that fits the data points (black line in Fig. 4) provide the values for log(N/Q) and log(e)/$T_{ex}$, from which MADCUBA derives N and $T_{ex}$, calculating Q ($T_{ex}$) as explained above.

### Blended Transitions of EtA.

We present in Fig. 3 the transitions of EtA with line intensities $T_{A}^{*}>$5 mK, as predicted by the LTE simulation described in the main text, that appear blended with emission from other molecular species already identified in the G+0.693 molecular cloud. The spectroscopic information of these transitions is shown in Table 2.

### Tentative Detection of Ketenyl (HCCO) toward G+0.693–0.027.

**Fig. 6.**
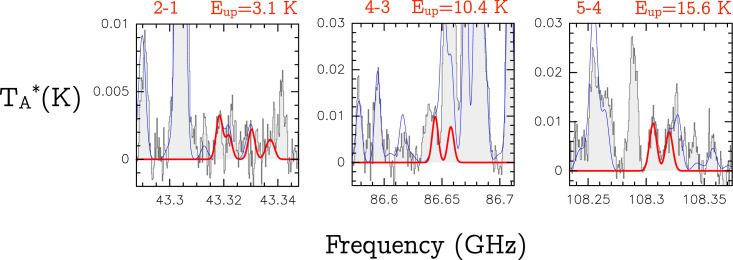
Transitions of HCCO tentatively identified in the spectra of G+0.693. The rotational quantum numbers involved in the transition are indicated in the upper left of each panel, and the energies of the upper level are indicated in the upper right. The thick red line depicts the LTE synthetic spectrum of HCCO. The thin blue line shows the predicted molecular emission from all of the molecular species identified in our spectral survey, overplotted to the observed spectra (gray histograms).

We have used the CDMS entry 041506 (June 2019), based on several spectroscopic works (65–67). We have tentatively identified three groups of HCCO lines corresponding to the rotational transitions 2−1, 4−3, and 5−4. The spectra are shown in Fig. 6, and the spectroscopic information of the transitions is listed in Table 3. This detection should be considered tentative, since only two transitions, the 5(6,6)−4(5,5) and 5(6,5)−4(5,4) (at 108.3040553 GHz and 108.3051187 GHz, respectively) are not contaminated by emission from other species (Fig. 6). We have produced LTE spectra using MADCUBA-SLIM and assuming $v_{LSR}$ = 65 km$\cdot s^{-1}$ and FWHM = 20 km$\cdot s^{-1}$. The predicted spectra reproduce well the two unblended transitions for a $T_{ex}$ of 10 K and a column density of $N∼0.5\times 10^{13}\;{cm}^{-2}$ (thick red line in Fig. 6). This column density translates into a molecular abundance of $∼0.4\times 10^{-10}$ with respect to molecular hydrogen.

**Table 3.** Spectroscopic information (rest frequency, Einstein coefficients [$A_{ul}$], and energy of the upper levels [$E_{up}$]) of the rotational transitions of ketenyl (HCCO) tentatively detected toward the G+0.693 molecular cloud (shown in Fig. 6)

Frequency, GHz	Transition	log$A_{ul}$, $s^{-1}$	$E_{up}$, K
43.3176674	2(3,3)–1(2,2)	−6.0192	3.1
43.3211451	2(3,2)–1(2,1)	−6.1404	3.1
43.3295421	2(2,2)–1(1,1)	−6.0343	3.1
43.3354627	2(2,1)–1(1,0)	−6.2739	3.1
43.3368615	2(3,2)–1(2,2)	−6.6741	3.1
43.3373040	2(2,1)–1(2,1)	−6.4207	3.1
86.6191857	4(4,3)–3(3,3)	−6.9202	10.4
86.6423419	4(5,5)–3(4,4)	−5.0703	10.4
86.6438483	4(5,4)–3(4,3)	−5.0942	10.4
86.6558306	4(4,4)–3(3,3)	−5.0772	10.4
86.6574849	4(4,3)–3(3,2)	−5.1070	10.4
86.6652791	4(5,4)–3(4,4)	−6.3845	10.4
108.2823800	5(5,4)–4(4,4)	−6.7293	15.6
108.3040553	5(6,6)–4(5,5)	−4.7698	15.6
108.3051187	5(6,5)–4(5,4)	−4.7840	15.6
108.3178903	5(5,5)–4(4,4)	−4.7747	15.6
108.3190248	5(5,4)–4(4,3)	−4.7916	15.6
108.3280559	5(6,5)–4(5,5)	−6.2997	15.6

## Data Availability

Molecular spectra and fits of the unblended transitions of ethanolamine have been deposited in the Centro de Astrobiologia repository at https://cab.inta-csic.es/astrochem/data.html (68).

## Footnotes

*Madrid Data Cube Analysis on ImageJ is a software developed at the Center of Astrobiology (CAB) in Madrid: https://cab.inta-csic.es/madcuba/.
